# Perspectives on Contraceptives: Insights From Care Providers and Women With Lived Experiences of Unintended Pregnancy in the Netherlands

**DOI:** 10.1111/psrh.70030

**Published:** 2025-08-20

**Authors:** Clair A. Enthoven, Hanan El Marroun, Simone Griesbergen, Femke Truijens, Hilmar H. Bijma, Pauline W. Jansen

**Affiliations:** ^1^ Department of Child and Adolescent Psychiatry/Psychology Erasmus University Medical Center Rotterdam the Netherlands; ^2^ Department of Psychology, Education and Child Studies Erasmus School of Social and Behavioural Sciences, Erasmus University Rotterdam the Netherlands; ^3^ The Generation R Study Group Erasmus University Medical Center Rotterdam the Netherlands; ^4^ Department of Obstetrics and Gynaecology, Division of Obstetrics and Fetal Medicine Erasmus MC Sophia Rotterdam the Netherlands; ^5^ Department of Care Ethics University of Humanistic Studies Utrecht the Netherlands

## Abstract

**Objectives:**

This study aims to develop a greater understanding of why unintended pregnancies happen by exploring views on contraceptive use of both care providers and women with lived experience of an unintended pregnancy in the Netherlands.

**Methods:**

We interviewed seven care providers involved in the care of women with unintended pregnancy in a focus group and individually interviewed 10 women with unintended pregnancy. We used reflexive thematic analysis.

**Results:**

Four themes emerged around the understanding of (lack of) contraceptive use in unintended pregnancies: “contraception perceived as unnecessary,” “psychosocial adversities,” “absence of suitable contraception,” and “failing contraception.” Among the care providers, the first two themes were most clearly present. Among women with unintended pregnancy, the last two themes were mainly emphasized.

**Conclusions:**

Women with unintended pregnancies mention a lack of suitable contraceptive options and failing contraception as important factors for unintended pregnancy, whereas care providers view the perception of contraception as being unnecessary and psychosocial adversity as important factors in unintended pregnancies. These differences point to the importance of explicitly asking about and addressing the perspectives of women in contraceptive counseling.

**Abbreviation**
IUD: intrauterine device

## 1 Introduction

The Netherlands has a long history of high rates of contraception users and low rates of unintended pregnancies [1, 2]. Yet, about 3% of Dutch men and women experienced an unintended pregnancy in 2016, and around 20% of the women during their lifetime [3, 4]. Furthermore, the use of contraception has decreased from about 66% of the sexually active 15‐ to 49‐year‐old women in 2017 to 59% in 2023 [3, 5]. Of those not using contraception, 32% mentioned not wanting to use hormones, 18% mentioned side effects, and 2% mentioned that contraception is too expensive [5]. Although these numbers seem useful for policy making, research has shown that decisions around contraception and family planning are complex [6], and a better understanding of the factors that shape contraceptive use is needed. Numbers on contraceptive use have traditionally been reported among women only, but we acknowledge that not only women may use contraception.

Ample research has focused on user preferences or care provider perspectives in contraceptive counseling [7, 8]. While research on user preferences recommends providers to prioritize client autonomy in decision‐making, research on the provider perspective has shown a paradox, where the priority of client autonomy is aligned with what they think is best for their client [8, 9]. Furthermore, the approach of research toward unintended pregnancy has recently been criticized for emphasizing too much on individual factors, such as personal choice, contraceptive use, and decision‐making, while overlooking the broader societal factors that influence reproductive choices [10]. For example, Mann et al. (2022) showed an unconscious bias among care providers informed by structural inequalities, which influence how patients are perceived and approached in contraceptive counseling [11]. Moreover, most research on contraceptive use includes either the user perspective [7, 12, 13, 14] or the care provider perspective [8, 15], while including both lived experiences and care providers' perspectives may be most valuable to inform relevant and achievable policies. Therefore, this study aims to develop a more comprehensive understanding of why unintended pregnancies happen by exploring views on contraceptive use of both care providers and women with lived experience of an unintended pregnancy.

## 2 Methods

This study employed a qualitative design using thematic analysis of a semi‐structured focus group interview with care providers and semi‐structured, in‐depth interviews with women with unintended pregnancy. A qualitative design was chosen because of the exploratory nature of the research question. Furthermore, a qualitative approach is particularly suitable for discussing sensitive multi‐faceted subjects [16]. We chose to interview both care providers and people with lived experiences to gain a greater, multi‐informant understanding of contraceptive use in unintended pregnancies.

The study took place between November 2021 and October 2022. Focus group participants were eligible if they provided care to people with unintended pregnancy. We recruited participants via our network and contacted them by email, and eight care providers agreed to participate. However, one care provider had to cancel due to illness, leaving seven focus group participants. The focus group took place online due to COVID restrictions. We set up the focus group interview with the aim to put participants at ease, to allow for an open sharing of views, and to discuss issues around contraceptive use. The care providers shared views and experiences on contraceptive use among people with unintended pregnancies by using a topic list (Table S1). The focus group interview took 1.5 h, and care providers received €50 reimbursement. A senior researcher (female gender, sociologist) led the focus group. Next to the participants and moderator, three other researchers were present (C.A.E., H.M., P.W.J., all female gender).

For the interviews on lived experience, participants were eligible for inclusion if they were over 16 years and had an unintended pregnancy at a maximum 2 years prior to the interview. Two persons were contacted and eligible but did not participate because they were too busy. We recruited the participants face‐to‐face during visits at the outpatient clinic of the Department of Obstetrics and Gynecology of the Erasmus University Medical Center, and via online social media. The participant decided the location for the interviews. We conducted semi‐structured, in‐depth interviews using a topic list, consisting of topics related to contraceptive use and pregnancy (Table S2). The interview guide also consisted of topics regarding experiences of care and parenthood as well as contraceptive use and pregnancy planning. The interviews took around 1 h, and participants received a €25 gift card upon interview completion. A senior researcher (C.A.E., female gender, health scientist) conducted the interviews, and two interns alternately joined some of the interviews. We asked about demographic information and psychosocial adversities in an open manner during the interview. We defined psychosocial adversity as psychosocial adversity that is associated with an increased risk of unplanned pregnancy and unfavorable outcomes of an unplanned pregnancy for women and their partners and the (future) child. This included mental illness, addiction, poverty, and/or lack of housing [17]. We included information as disclosed by the participants.

We used purposive sampling aiming for maximum variation of psychosocial adversity. Recruitment was stopped after the research team reached data saturation as determined by a lack of emergence of new codes on the main themes. After analyzing multiple transcripts, the recurring themes became consistent and we concluded that data saturation had been achieved [18, 19].

All participants provided oral and written informed consent for study participation. The Ethics Review Committee of the Department of Clinical Psychology, Education and Child Studies, Erasmus University Rotterdam provided ethical approval (ETH2021‐0109). Data are reported using the COREQ criteria [20].

### 2.1 Thematic Analyses

We audio‐recorded the focus group interview and individual interviews and transcribed verbatim. We did not return the transcripts to participants. During the focus group interview, we took some field notes, but only to help guide the discussion. We used reflexive thematic analysis by Braun and Clarke for analysis of the transcripts [21]. Two researchers (C.A.E. health scientist and S.G. clinical psychologist, both female gender) coded expressions relating to contraception and pregnancy planning. Then, they created overarching themes using the codes of constructing themes and reflected on the meaning of these themes. Since this is an iterative process, the researchers went back and forth between coding the transcripts and constructing the themes after each interview. After having created themes for all interviews, they used triangulation by comparing the themes emerging from the focus group interview and from the individual interviews. In case differences between the two researchers arose, they discussed the coding and the themes until agreement was reached [22]. We used the software Atlas.ti for the thematic analysis [23]. We enhanced reflexivity by discussing the results at regular meetings with a senior researcher in qualitative research (H.B.).

## 3 Results

Table 1 shows the characteristics of all participants. Care providers 1, 2, and 3 were staff involved in family planning at a public health organization funded by the municipality. This organization, aiming to support women and men with psychosocial adversity, provides counseling around family planning, funding of contraception, and practical support, such as reminding of appointments and accompanying at medical contraception appointments. Care provider 4 is a health psychologist providing counseling to pregnant people in vulnerable circumstances (i.e., financial problems, a disadvantageous lifestyle, or the avoidance of care) [24]. Care provider 5 is a hospital‐based midwife who previously worked as a community midwife. Care providers 6 and 7 are general practitioners who both regularly prescribe contraception (Table 1).

**TABLE 1:** Participants of focus group and individual interviews.

Care providers	Position of care provider	Domain
1	Sexologist, public health nurse	Municipal health services
2	Content coordinator/pedagogue	Municipal health services
3	Content coordinator/social nurse	Municipal health services
4	Health psychologist and pedagogue	Hospital, department of psychiatry
5	Hospital‐based midwife	Hospital, department of obstetrics and gynecology
6	General practitioner	General practice
7	General practitioner	General practice

Participants with unintended pregnancy	Age at pregnancy	First pregnancy	Previous unintended pregnancy	Reproductive stage	Relationship	Psychosocial adversity
1	30	No	Yes, continued	Third trimester	Yes	Yes, multiple
2	30	Yes	No	Child 0–2 years	No	Yes, single
3	25	No	Yes, continued	Child 0–2 years	Yes	None known
4	29	Yes	No	Child 0–2 years	Yes	None known
5	29	Yes	No	Child 0–2 years	Yes	None known
6	35	No	No	Child 0–2 years	Yes	None known
7	22	Yes	No	Child 0–2 years	No	Yes, multiple
8	32	Yes	No	Child 0–2 years	No	Yes, multiple
9	38	No	Yes, abortion	Third trimester	Yes	Yes, single
10	25	Yes	No	Child 0–2 years	No	None known

Among the participants with lived experience, further referred to as “women,” seven women were interviewed at home, two online, and one in the hospital. Six women were known with mental illness, three women had socio‐economic adversity (e.g., poverty, lack of income, or lack of housing), and two women were known with substance use disorder during pregnancy (Table 1).

We found four main themes emerging from the interviews. Two themes that most clearly emerged in the interviews with women with unintended pregnancy reflected the properties of the contraceptive methods themselves and can be captured as “absence of suitable contraception” and “failing contraception.” The two other emerging themes were most clearly described by care providers as important in the context of unintended pregnancy. These themes reflected “contraception perceived as unnecessary” and “psychosocial adversity.”

### 3.1 Absence of Suitable Contraception

So, actually I'm still looking for the best thing to do now, because there is no such thing without hormones and something that is not an IUD, except condoms. That is also possible, but it is less practical of course. (Woman 3)

This theme describes how a perceived lack of suitable contraception is mentioned by women as a reason for not using contraception and for their unintended pregnancy. Women 3, 5, 6, 7, 9 and 10 decided to stop using hormonal contraception because they attributed emotional side effects to it. They experienced a decreased mood and read or heard from others that hormonal contraception may be related to mood swings. Woman 7 described how the impact of hormonal contraception on her mood resulted in perceiving hormonal contraception as something unfavorable: *“And because it flattened my emotions so much I thought, this can't be good, so I didn't take anything [hormonal contraception] anymore.”* Additionally, women 1, 7, and 9 experienced an unfavorable physical impact. They reported a feeling of being pregnant, a negative influence on symptoms of chronic conditions, pain during intercourse, spotting and irregular periods. For instance, woman 9 shared: *“I also noticed a lot of difference in my stomach when all those hormones were out of my body.”* Finally, women 3, 4, and 8 experienced a limited choice of contraceptive methods because of medical reasons. They shared that care providers advised them against using hormonal contraception and/or the copper IUD due to contra‐indications. Woman 4 shared: *“I do not use hormones or anything in terms of contraception because I have had a pulmonary embolism in the past. So I was advised not to do that.”* Hence, nine women with unintended pregnancy perceive a lack of suitable contraception which they attribute to properties of contraceptive methods or lack of suitable contraception in their specific medical situation.

In contrast with the experiences of women with unintended pregnancies, the interviewed care providers attributed a perceived lack of suitable contraception to misconceptions or lack of knowledge among women. For example, care provider 1 shared her view on a misconception of the impact of hormonal contraception on weight: “*I had a client who said I'm getting thin from it […] when I further asked, it turned out that there was another problem that caused her to lose weight*.” Additionally, care providers shared how a sense of contraception not being natural may result in non‐use: *“The group who consciously does not want contraception are, in my opinion, the more highly educated women who read all kinds of things about the Moonsisters [A community of women that believe that lunar cycle has impact on human reproduction, menstruation, mental health and mood] and similar groups on the internet, and recognize themselves in them.”* (care provider 7). Finally, care providers shared their perceptions on how women refrain from using contraception from overgeneralizing other people's experiences with contraception: *“We also see vulnerable people who do not want to use hormones […] who have little adequate information and therefore do not want to use them, or ‘my mother got sick from it, so it is not good for me either’”* (care provider 1). This is in line with an overgeneralizing perception around contraception that woman 1 shared: *“So I thought, I'm going to use the implant, but my cousin said no, because she had a bad experience with the implant.”* Remarkably, women who had already delivered after an unintended pregnancy, still struggled with contraceptive choices, with women 3 and 5 not using contraception at the time of the interview. Whereas woman 3 actively consulted a gynecologist for advice, woman 5 did not seek for help with finding suitable contraception, because of being taken up by a busy life.

### 3.2 Failing Contraception

One of the themes that emerged was “failing contraception,” which describes that women got pregnant despite (intended) use of contraception and in absence of wanting to conceive. Woman 3 described a failure of contraception out of her reach, as she described falling pregnant despite strict use of oral contraception: *“I had my alarm set for 10 pm every day […] I always took my pill, so I was very strict about it.”* Women 1, 4, 5, 6, 8, and 9 described falling pregnant because their behavior did not match their intention for using contraception such as condoms, (periodic) abstinence or a combination of the two. Some once forgot to use a condom, because they just had not thought of it, or because the sex was unexpected, as described by woman 10: *“Because I actually didn't expect that I would have sex […] it was kind of an unexpected moment and then you get a little caught up in the moment and then you just don't think that much anymore.”*

Care providers attributed failing contraception to inconsistent use of contraception, which they attributed to a lack of structure, agency and/or knowledge. In their opinion, short‐term methods, such as oral contraception, the contraceptive patch or ring, are not suitable for everyone. Care provider 1 shared: *“If you have a reversed day‐night rhythm, if you live in a car, if you don't have a toothbrush and no sink where you normally put it, then taking the pill is quite complicated.”* They also shared that many women with unintended pregnancies seem to live in the present and do not think about the consequences of having unprotected sex. Care provider 5 indicated: “*Sometimes just because it was raining and the pregnant woman did not feel like going outside to get condoms*.” Finally, the care providers shared that some of their clients or patients became pregnant due to incorrect use of contraception, inaccurate information, or not knowing when they were fertile. Care provider 1 explained: *“And I think, a lot of, at least of our clients, they don't really know exactly when am I actually fertile and when am I at risk.”* None of the women explicitly mentioned that (lack of) knowledge played a role in their contraceptive use.

### 3.3 Contraception Perceived as Unnecessary

Many clients we see, feel something like ‘I'm a women so I'm just going to have children’ […] But it's actually also that you can choose whether or not you get pregnant, which is not really the case for a lot of people. (Care provider 4)

A third theme that emerged was how contraception was perceived as unnecessary, because of not having a partner or because of perceiving the chance of falling pregnant as very low.

Both the care providers and the women mentioned not being in a relationship as a reason for not using contraception. Woman 2 and 4 both shared that after they broke up with their partners, they did not feel the need to find suitable contraception. Care provider 1 shared: *“Such a partner always comes suddenly, so then it's not prepared.”*

In addition, both care providers and women mentioned infertility. Some care providers (# 4 and 5) mentioned that a pregnancy is very unlikely for some women with medical conditions. As these women do not expect to become pregnant, it is often unintended when it happens. In line with this, woman 7 shared that doctors had told her that she was infertile due to a medical condition. However, she did not relate this to contraceptive use or her unintended pregnancy, as she fell pregnant due to rape. Other care providers (# 1 and 5) mentioned experiences of clients who had unprotected sex for years without becoming pregnant, and then assumed that they were infertile. Or clients with fertility problems during a first pregnancy, and then unexpectedly became pregnant of a second child. Care provider 1 shared: *“And then they think […] I must be infertile, so I don't need birth control at all.”* Indeed, women 1, 5, 6, and 8 shared how they did not use contraception from the perception that the chance of pregnancy is generally quite low. They expressed disbelief that they could get pregnant from having unprotected sex once (# 1, 6, and 8), because they experienced problems with trying to get pregnant in the past (# 8), or they knew couples who had difficulties with getting pregnant (# 5). Woman 8 shared: *“I just really didn't believe it. I thought, how is that possible in one go [one time unprotected sex]?”* Also, after having unprotected sex a few times without getting pregnant, women 1 and 6 continued to use less reliable methods. Woman 1 shared: *“Yes, it was one, two, three months [without pregnancy] and I thought it's going well.”*

Care providers also mentioned that some, particularly young, women may have a latent desire to become mother and therefore do not use contraception. Perhaps the pregnancy is then “semi‐unintended,” with a care provider describing that in these cases, the care provider may find it more unintended than the client. “*It's not always a problem for themselves, it's more that the care providers around them think eh … yeah … this could have been planned better*” (care provider 2). This was also reflected in the stories of women 2 and 8 who both desired children with a partner but participant 2 had a difficult relationship, and participant 8 did not have a partner. Participant 2 shared: *“And that night I told him the only way we can share the bed together is when you're really ready for the possibility that I'll become pregnant. And he promised: ‘yes, yes, yes’.”* Finally, care providers 3 and 7 shared their experience that some women do not have the feeling that they have a choice in reproduction. Care provider 3 explained: *“Some women think ‘well, getting pregnant is just part of being woman’.”* None of the women mentioned this.

### 3.4 Psychosocial Adversities

We almost always see a combination of factors. Indeed, psychological problems, debt problems, addiction problems or being undocumented and financial problems or lack of access [to healthcare]. It's almost never just one thing. There are always several things at once. (Care provider 1)

Factors indicating psychosocial adversity were mainly described by care providers. First, some care providers (# 1, 4, and 6) mentioned that unintended pregnancy may be the consequence of a coercive relationship, domestic violence or rape. They noticed that the partner sometimes pressures the woman to become pregnant, for example by forcing her to accept intercourse without a condom. Woman 7 shared that she became pregnant due to rape by her ex‐partner. She shared: *“My neighbor didn't want to take me home because she was also drunk, then I ended up going with my ex, I should never have done that actually. And then I got pregnant in a violent way at that moment. He just raped me.”* Second, the care providers described that some people are financially not able to purchase contraception. Care provider 1 shared: “*Because it is no longer included in the basic health insurance above the age of 21. There are also people who have tried to prevent it [unintended pregnancy], but it is not possible because they do not have the financial means to pay for contraception*.” Even though some women indicated to have financial difficulties, none of them related this to their contraceptive use or unintended pregnancy. Third, the care providers (# 1, 2, 4, and 6) shared that mental health problems, such as trauma, addiction and cognitive impairment are common among women with unintended pregnancies. Care provider 4 explained: *“The core is really psychological problems or psychiatric history, with childhood trauma or pregnancy related trauma.”* They related psychiatric adversity to other subthemes, such as “latent desire for children,” “lack of knowledge,” or “contraceptive inconsistency.” Some women (# 1, 2, 7, 8, and 9) indeed shared they have had mental illnesses, but none of them related this to their contraceptive use or unintended pregnancy. Finally, several care providers (# 1, 2, 3, 5, and 6) mentioned that young girls experience barriers to get contraception because their parents may not know that they are sexually active, or they have a difficult home situation in which they lack a support system. Care provider 6 shared: “*And if you just have other sixteen year olds as your support system, you don't get triggered [to start contraception] by that*.” Some women were relatively young and explained having a difficult childhood, but they did not relate this to their contraceptive use or unintended pregnancy.

## 4 Discussion

In this study, we developed a greater understanding of why unintended pregnancies happen by exploring views of both care providers and women with lived experience of an unintended pregnancy. We found several themes related to contraceptive use and unintended pregnancy. First, women with unintended pregnancies experienced problems finding suitable contraception due to perceived emotional or physical side effects, having a limited choice because of medical reasons and/or resistance against (hormonal) contraception. Second, women also related their unintended pregnancy to failing contraception while on hormonal contraception, or inconsistent use of contraception. Both of these themes were also mentioned by care providers. In contrast, the remaining two themes were more emphasized by care providers and less by women with lived experience of unintended pregnancy. In the third theme that emerged, care providers indicated that contraception is sometimes perceived as not needed because there is no current partner, (perceived) infertility, a latent desire for children, or a lack of choice in whether or not to conceive. Finally, the fourth theme encompassed factors related to psychosocial adversity, that is, financial constraint, mental problems, young age, and relationship problems, that were also mentioned mainly by care providers.

Some limitations of this study should be considered before interpreting the findings. First, the lack of agreement between the care providers and women in some of the sub‐themes may partly be due to sampling bias. We recruited the women with lived experience via the outpatient clinic and via online social media. We used purposive sampling aiming at maximum variation of psychosocial adversity. While studies often have difficulties with including participants with psychosocial adversities, we were able to include participants who experienced addiction, severe psychiatric problems, or homelessness via the outpatient clinic. However, there are many different psychosocial adversities, and it was beyond the scope of this study to have all situations reflected in our sample. Some women with lived experience were recruited via online social media, which may have led to disproportionately including individuals who are more digitally literate or motivated to share their experiences. Since we recruited the care providers through our own professional network, most of them were working with individuals facing psychosocial adversities in the city of Rotterdam. As a result, these care providers may be more familiar with this target group, and therefore may not be fully representative of all care providers in the Netherlands. Second, we only included participants who decided to continue the pregnancy, while lived experiences of those who decided to terminate the pregnancy may be different from those who continued the pregnancy. Finally, this study focused on cisgender women only, while men and gender diverse populations also use contraception. Their experiences with contraception and unintended pregnancy may be very different from cisgender women [25, 26].

The care providers shared their perspectives that psychosocial adversities may sometimes lead to unintended pregnancies. Other studies, including our own [27], showed a co‐occurrence between financial hardship, childhood trauma, and mental health problems among people with unintended pregnancy [28, 29]. However, patients described as “vulnerable” may only accept the label when the challenges they have faced in the past are also acknowledged and their strength in overcoming these obstacles and seeking support is seen [30]. It is also important to recognize that such adversities are not intrinsic but rather the product of our societal context with its oppressive systems, including classism, racism, and sexism [31, 32]. This should be kept in mind while determining intervention efforts that may assist individuals. Suggested mechanisms that may explain the link between psychiatric adversity and unintended pregnancy are lack of planning capacities, poor compliance with contraceptive methods, or a perceived low risk of pregnancy associated with unprotected sex [28, 33]. The women in our study did not link their financial difficulties, psychiatric history, or other adversities to their contraceptive use, but the suggested mechanisms that may potentially underlie this link were part of other themes shared by them.

Another important finding of this study is that some women perceived currently available contraceptive methods as not suitable for them. Some women expressed resistance against hormonal contraception because of mental or physical side effects. They therefore used less reliable methods such as condoms, periodic abstinence, or a combination of both. A recent systematic review showed that hormonal contraception was often rejected due to mental and physical health problems, decreased sexual desire, concerns, and fear over future fertility and over hormones being unnatural, as well as irregular menstruation [34]. Additionally, in line with the results from our study, worries about adverse side effects were not always taken seriously by care providers [34, 35]. Ideal contraception should be reliable, affordable, and easy to use, and that does not cause any undesired physiological side effects [36]. Despite having a large variety of available options, some individuals will likely not find a method that has all features that they find important [36, 37, 38]. This may lead to discontinuation or switching contraceptive methods, including gaps in between different methods which increase the risk of unintended pregnancy [39].

The results of this study show that it is important for care providers to realize that there may not only be a knowledge gap between them and women with unintended pregnancy, but also a gap in lived experience. Hence, care providers are encouraged to become sufficiently aware of the personal situation and history of women in child‐bearing ages, to be able to take into account those conditions and their role in contraception use. The women emphasized their (dis)satisfaction with reliable contraceptive methods, and therefore changed to less reliable methods or no methods at all in case they did not have a partner, had a latent desire for a pregnancy, or perceived a low risk of a pregnancy. From their lived experiences, dissatisfaction with contraception was in the first place the key element, which must be taken seriously by care providers. However, only one woman consulted a care provider for alternative methods, suggesting that some participants may experience limited contraceptive agency [40].

In conclusion, the results of this study show differences in perspectives between care providers and women with lived experiences of unintended pregnancy about factors that shape contraceptive use. Care providers emphasized that psychiatric and socioeconomic adversities may limit women's agency in using contraception effectively, while the women mainly highlighted the lack of suitable contraceptive options, leading them to rely on less effective methods. This underscores the importance of taking worries about side effects and other issues seriously to improve contraceptive care. Shared themes between care providers and the women included not needing contraception due to the absence of a partner, and a latent desire for children, underscoring the value of open discussions between care providers and clients about family planning.

## Conflicts of Interest

The authors declare no conflicts of interest.

## Supporting information


**Data S1:** Supporting Information.

## Data Availability

The data that support the findings of this study are available on request from the corresponding author. The data are not publicly available due to privacy or ethical restrictions.
