# Supplementary material for: Perspectives on Contraceptives: Insights From Care Providers and Women With Lived Experiences of Unintended Pregnancy in the Netherlands
Source: Perspect Sex Reprod Health. 2025 Aug 20;57(3):350–7. doi: 10.1111/psrh.70030 (PMC12421086; doi:10.1111/psrh.70030)
Supplement: Supplementary file 1 — Data S1: Supporting Information. [file PSRH-57-350-s001.docx]

**Supplemental material**

**Table S1: The topic guide for the focus group interview**

| **Introduction round** |
| --- |
| **Opening question**  How do you encounter unplanned pregnancies in your daily work? |
| **Theme 1: Causes of unplanned pregnancies**   1. What are causes of unplanned pregnancies? 2. Which are most important? |
| **Theme 2: Risk factors for unplanned pregnancies**   - What do you consider risk factors for unplanned pregnancies? - Which factors are specific to women and which factors are specific to men? - Which risk factors often occur together? - Are there any risk factors associated with not using contraception or not using contraception properly? - Which factors are underexposed in the professional field or in society? |
| **Theme 3: Prevention of unplanned pregnancies**   - What is already being done in prevention? - What is still needed or what can be improved? |
| **Theme 4: Required knowledge in the field**   - What knowledge do you think is still needed? - Which questions would you like to have answered yourself? |
| **Closure**  Is there anything important that you feel has not been discussed? |

**Table S2: The topic guide for the semi-structured interviews**

| **Opening question**  Can you tell me your story? What your life was like before you got pregnant; How was the moment you found out you were pregnant and how are you doing now? |
| --- |
| **Unintended pregnancy**   - Timing of the pregnancy - Wantedness - Use of contraception - Previous pregnancies |
| **Feelings toward the pregnancy & partner/father of the child**   - Do you currently have a partner?/How is your relationship with the father? - What did the partner/father of your child think of the pregnancy? - How did you find you that you were pregnant? What was your first thought? - Did these feelings change during the pregnancy? - How did your environment react about the pregnancy? |
| **Lifestyle prior to and during pregnancy**   - Nutrition, vitamin pills (including folic acid), medication, physical activity, and substance use prior and during pregnancy |
| **Finances and practical help**   - Life prior to pregnancy (work/study) - How did the work/study environment react to your pregnancy? - Financial hardship - Practical or financial support from family or social network |
| **Professional care**   - Experiences around obstetric care - Other social, psychological, or health care - Unmet need for additional professional (health) care |
| **Expectations/experience of motherhood**   - What did you look forward too? - What challenges did you foresee? - How are you doing now? |
| **Closure**   - Is there something you would like to add? |
